# Effect of Monochromatic Light on Expression of Estrogen Receptor (ER) and Progesterone Receptor (PR) in Ovarian Follicles of Chicken

**DOI:** 10.1371/journal.pone.0144102

**Published:** 2015-12-01

**Authors:** Lingbin Liu, Diyan Li, Elizabeth R. Gilbert, Qihai Xiao, Xiaoling Zhao, Yan Wang, Huadong Yin, Qing Zhu

**Affiliations:** 1 Institute of Animal Genetics and Breeding, Sichuan Agricultural University, Ya’an, Sichuan, P. R. China; 2 Department of Animal and Poultry Sciences, Virginia Tech, Blacksburg, Virginia, United States of America; 3 College of Animal science and technology, Sichuan Agricultural University, Ya’an, Sichuan, P. R. China; University of Wisconsin - Madison, UNITED STATES

## Abstract

Artificial illumination is widely used in modern poultry houses and different wavelengths of light affect poultry production and behaviour. In this study, we measure mRNA and protein abundance of estrogen receptors (ERs) and progesterone receptors (PRs) in order to investigate the effect of monochromatic light on egg production traits and gonadal hormone function in chicken ovarian follicles. Five hundred and fifty-two 19-wk-old laying hens were exposed to three monochromatic lights: red (RL; 660 nm), green (GL; 560 nm), blue (BL; 480 nm) and control cool white (400–760 nm) light with an LED (light-emitting diode). There were 4 identical light-controlled rooms (n = 138) each containing 3 replicate pens (46 birds per pen). Water was supplied ad libitum and daily rations were determined according to the nutrient suggestions for poultry. Results showed that under BL conditions there was an increase in the total number of eggs at 300 days of age and egg-laying rate during the peak laying period. The BL and GL extended the duration of the peak laying period. Plasma melatonin was lowest in birds reared under BL. Plasma estradiol was elevated in the GL-exposed laying hens, and GL and BL increased progesterone at 28 wk of age. In the granulosa layers of the fifth largest preovulatory follicle (F5), the third largest preovulatory follicle (F3) and the largest preovulatory follicle (F1), ERα mRNA was increased by BL and GL. Treatment with BL increased ERβ mRNA in granulosa layers of F5, F3 and F1, while GL increased ERβ mRNA in F5 and F3. There was a corresponding increase in abundance of the proteins in the granulosa layers of F5, with an increase in PR-B, generated via an alternative splice site, relative to PR-A. Treatment with BL also increased expression of PR mRNA in all of the granulosa layers of follicles, while treatment with GL increased expression of PR mRNA in granulosa layers of SYF(small yellow follicle), F5 and F1. These results indicate that blue and green monochromatic lights promote egg production traits via stimulating gonadal hormone secretion and up-regulating expression of ERs and PRs. Changes in PR-B protein suggest that this form of the progesterone receptor is predominant for progesterone action in the granulosa layers of preovulatory follicles in chickens during light stimulation.

## Introduction

Artificial illumination is widely used in modern poultry houses. However, the intensity, duration, and quality of light are important environmental factors influencing chicken reproductive and productive systems [1]. The chicken eye has two types of receptor cells in the retina: rods and cones. The rods allow vision in poor light. In contrast, the cones are responsible for normal daytime vision [2, 3]. Unlike humans (three types) [2], there are four types of cones in the retina of the chicken eye that have more nerve connections between the photoreceptors and the brain [4, 5]. These traits give birds the ability to perceive not only the human-visible range of light but also the ultraviolet part of the spectrum, as well as allowing for the detection of polarized light and magnetic fields [6, 7].

Although colour has been confounded with illuminance in many reports, different wavelengths of light have an unquestionable effect on poultry production and behavior [2]. Skoglund et al [8] found that broilers reared under blue (BL) or green light (GL) gained more weight than those exposed to red (RL)or cool white light (control group, CL), while Cave et al [9] showed that GL might increase chick production in broiler breeders by causing decreased pullet mortality and increased fertility. Er et al [10, 11] showed that egg weight in RL houses was less than in other lighted conditions, and the egg quality under GL conditions was superior to other lighting. Furthermore, Rozenboim [12] demonstrated that GL stimulated growth at an early age, and shifting birds to a different light environment might further stimulate growth. In another study, GL enhanced development and growth in chicks with the most pronounced effect achieved when the stimulus was provided during incubation [13]. In addition, Cao et al [14] suggested that GL and BL promoted growth and development of broilers via stimulating testosterone secretion and skeletal myofiber growth. These results show that monochromatic light influences poultry production and suggest a possible mechanism for the improvement in egg production traits associated with light stimulation.

The growth and development of ovarian follicles undergo a series of complex biochemical and physiological changes, which include hormone receptor expression, steroid biosynthesis, cell proliferation, and differentiation [15]. Melatonin is important hormone signal during illumination influencing chicken reproductive system [16]. The steroid hormone estrogen plays a profound role in regulating the structure and function of many target tissues in female reproductive systems. These include the mammary gland, ovary, uterus, vagina, and prostate [17]. There are two different forms of the estrogen receptor, usually referred to as α (ERα) and β (ERβ), each encoded by a separate gene [18]. Progesterone regulates reproductive function through two intracellular receptors, progesterone receptor–A (PR-A) and progesterone receptor–B (PR-B), that arise from a single gene and function as transcriptional regulators of progesterone-responsive genes [19].

We hypothesized that compared with red light, blue and green lights are associated with increasing reproductive system in laying hens. In this study, we measure mRNA and protein abundance of ERs and PRs in order to investigate the effect of monochromatic light on egg production traits and gonadal hormone function in ovarian follicles of Chinese local Erlang Mountainous Chickens, and it will provide scientific data for poultry lighting system.

## Materials and Methods

### Populations and Management

All animal protocols were approved by the Institutional Animal Care and Use Committee at Sichuan Agricultural University (No. DKY-B20110108). Five hundred and fifty-two19-wk-old Erlang Mountainous laying hens were randomly exposed to one of four light treatments (n = 138) in three replicate groups (n = 46): blue (BL, 435~500 nm), green (GL, 500~565 nm) and red (RL, 630~780 nm) light produced by light-emitting diode (LED) lamps, as well as cool white light (control, CL). Hens were exposed to treatments from 19 to 43 wk of age [2, 10, 20]. Birds were housed in individual laying batteries [25 cages (length × width× height = 50 cm × 38 cm × 35 cm), with 2 laying batteries per replicate]. During the first week (19 w), the light schedule was maintained at 13 h of light each day, increasing in increments of 0.5 h per week until 16 hours (L:D = 16 h:8 h) at 25 wk. All light sources were equalized to a light intensity of 15 lx (1.4 foot candle) [21]. Room temperature was measured daily, and each room had an environmental control system to maintain the temperature between 22 and 24°C. Water was supplied ad libitum and daily rations were determined according to the nutrient suggestions for poultry [22]. All hens were raised in the poultry breeding farm of Sichuan Agricultural University (Ya’an, Sichuan Province, China). The Erlang Mountainous Chicken originated from local chicken breeds in the Sichuan province [23–25]. We recorded the total number of eggs at 300 days of age (EN300), peak laying period (PLP), and egg-laying rate during the PLP. The egg-laying rate during the PLP was determined as follows: egg-laying rate during PLP = total number of eggs during PLP / (number of hens × number of PLP days)[15].

### Plasma concentrations of Melatonin, Estradiol and Progesterone

At 28 wk of age, 2 mL of blood was collected by venipuncture from five birds that were randomly selected from each group. 28 wk of age laying hens are during peak laying period. Plasma was immediately separated by centrifugation at 3000 x rpm for 15 min, and stored at -80°C. Plasma melatonin, estradiol and progesterone was measured with a chicken Melatonin, Estradiol-17β (E2) and Progesterone ELISA (Enzyme Linked Immunosorbent Assay) Kit (Gersioninc, Beijing, china), according to the recommendations of the manufacturer.

### Tissue Sampling

At 28 wk of age, five birds were randomly selected from each group and euthanized by cervical dislocation for collection of granulosa layers of preovulatory follicles [small yellow follicle (SYF), the fifth largest preovulatory follicle (F5), the third largest preovulatory follicle (F3) and the largest preovulatory follicle (F1)], classified according to Onagbesanet al [26]. All samples were wrapped in foil and snap-frozen in liquid nitrogen and stored at −80°C.

### Real-time PCR assays

Total RNA was isolated from the frozen tissues with Trizol reagent (TaKaRa, Dalian, China). The concentration and purity of RNA was determined by the A260/280 absorbance ratio (1.8–2.0), and the integrity of the 18S and 28S rRNA bands on a 2% agarose gel. First-strand cDNA was synthesized from 1μg total RNA using the PrimeScript^®^ RT reagent Kit Perfect Real-Time (TaKaRa, Biotechnology Co. Ltd., Dalian, China) following the manufacturer’s instructions. Reactions were performed under the following conditions: 42°C for 2min, 37°C for 15 min, and 85°C for 5sec and stored at 4°C.

Real-time PCR primers were designed by Primer Premier 5 (S1 Table). The PCR was carried out in a CFX96 (Bio-Rad, Inc., Richmond, CA, USA) qPCR system [27] and performed in triplicate in reaction volumes of 15 μL that included 1 μL of cDNA, 0.6 μL of forward and reverse primers (10 μM) for each gene, 7.5 μL of 2×SYBR green SuperMix (Bio-Rad, Inc., Richmond, CA, USA), and 5.3 μL of double-distilled H2O. The cycling conditions were as follows: 95°for 30s, 40 cycles of 95°for 5s and annealing temperature (β-actin: 61°, ERα: 53.3°, ERβ: 52.7°and PR: 56.9°) for 30s, and 95°for 10s, with a melt curve analysis performed at 65°~95°. The amplification efficiencies of ERs, PR and β-actin ranged from 95% to 105%.

### Protein Extraction and Western Blotting

The granulosa layers of F5 were used for protein isolation with the BSP003 kit (Sangon Biotech Co., Ltd, Shanghai, China). Protein concentration was measured with the Pierce bicinchoninic acid (BCA) Protein Assay Kit (Thermo Scientific Pierce, Rockford, IL, USA) and Varioskan Flash instrument (Thermo Fisher Scientific, Rockford, IL, USA). A total of 30 μg protein was resolved by sodium dodecyl sulfate-polyacrylamide gel electrophoresis (SDS-PAGE) and transferred to polyvinylidene fluoride (PVDF) membranes. After blocking with 5% non-fat milk in 1× Tris-Buffered Saline with Tween (TBST) buffer for 1 h at room temperature, membranes were incubated with rabbit anti-chicken (ERα, ERβ, PR-A, and PR-B) monoclonal antibodies (Abcom, Cambridge, UK, 1:1000) and rabbit anti-chicken β-actin monoclonal antibody (Abcom, Cambridge, UK, 1:1000) overnight at 4°C [27–29]. The blots were then washed in 1× TBST buffer and probed with goat-anti-rabbit horseradish peroxidase (HRP)-conjugated IgG secondary antibody (diluted 1:2000 in 1× TBST; Abcom, Cambridge, UK) for 1 h at room temperature. Binding was visualized with enhanced chemiluminescence (ECL) reagent (Beyotime Institute of Biotechnology, Jiangsu, China) using a ChemiDoc XRS instrument (Bio-Rad, Inc., Richmond, CA, USA). Quantity One Software (Bio-Rad, Inc., Richmond, CA, USA) was used for densitometric analysis.

### Statistical analyses

Statistical analysis was performed with the SAS 9.3 software (SAS Institute Inc., Cary, NC). All data are presented as means ± standard deviation, and analyzed for statistical significance by the Univariate General Linear Model followed by the LSD Multiple Comparison test for pairwise comparisons. Values differ significantly at P < 0.05.

## Results

### Effect of monochromatic light on production traits

**Fig 1:**
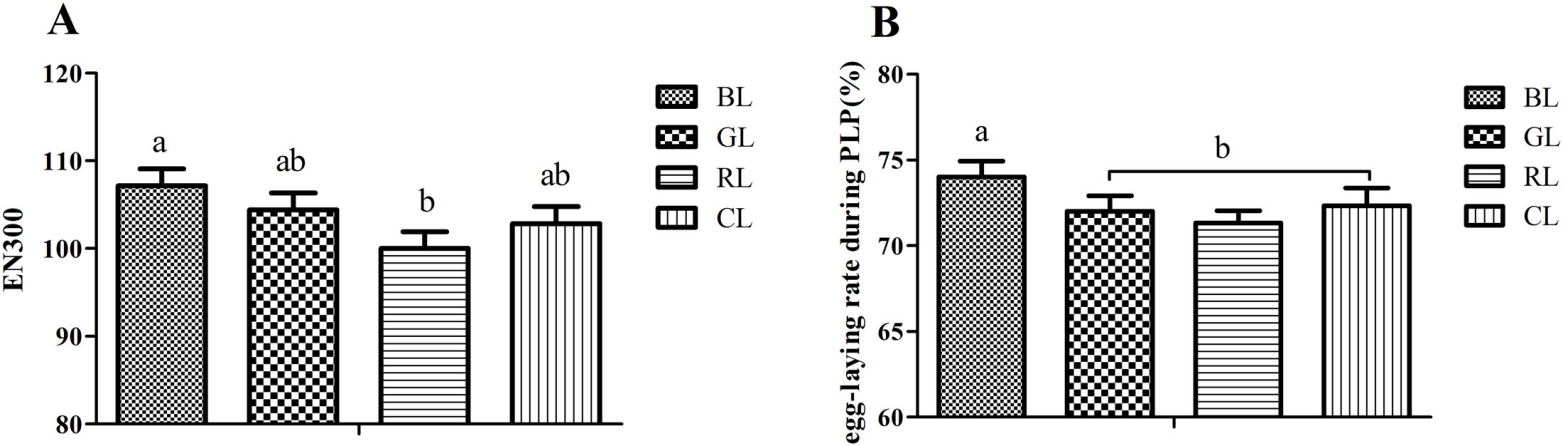
Effect of monochromatic light on EN300 and laying rate in PLP (%). EN300 = the total number of eggs at 300 days of age; egg-laying rate during PLP = egg-laying rate during the peak laying period. BL = blue light, GL = green light, RL = red light, and CL = cool white light (control group). Results are expressed as mean ± SD (n = 150). Least square means with different letters are significantly different (*P* < 0.05).

Effects of monochromatic light on production traits are summarized in Fig 1 and S2 Table. Birds reared under BL (blue light) increased the total number of eggs at 300 days of age (EN300, P < 0.05) (Fig 1A). BL and GL (green light) groups had an extended peak laying period (S2 Table), with the following rank order: GL (25–33 wk) > BL (26–34 wk) > CL (cool white light, 26–33 wk) > RL (red light, 28–33 wk). The egg-laying rate of BL chickens during the peak laying period was greater than other groups (Fig 1B).

### Effect of monochromatic light on plasma concentrations of melatonin, estradiol and progesterone

**Fig 2:**
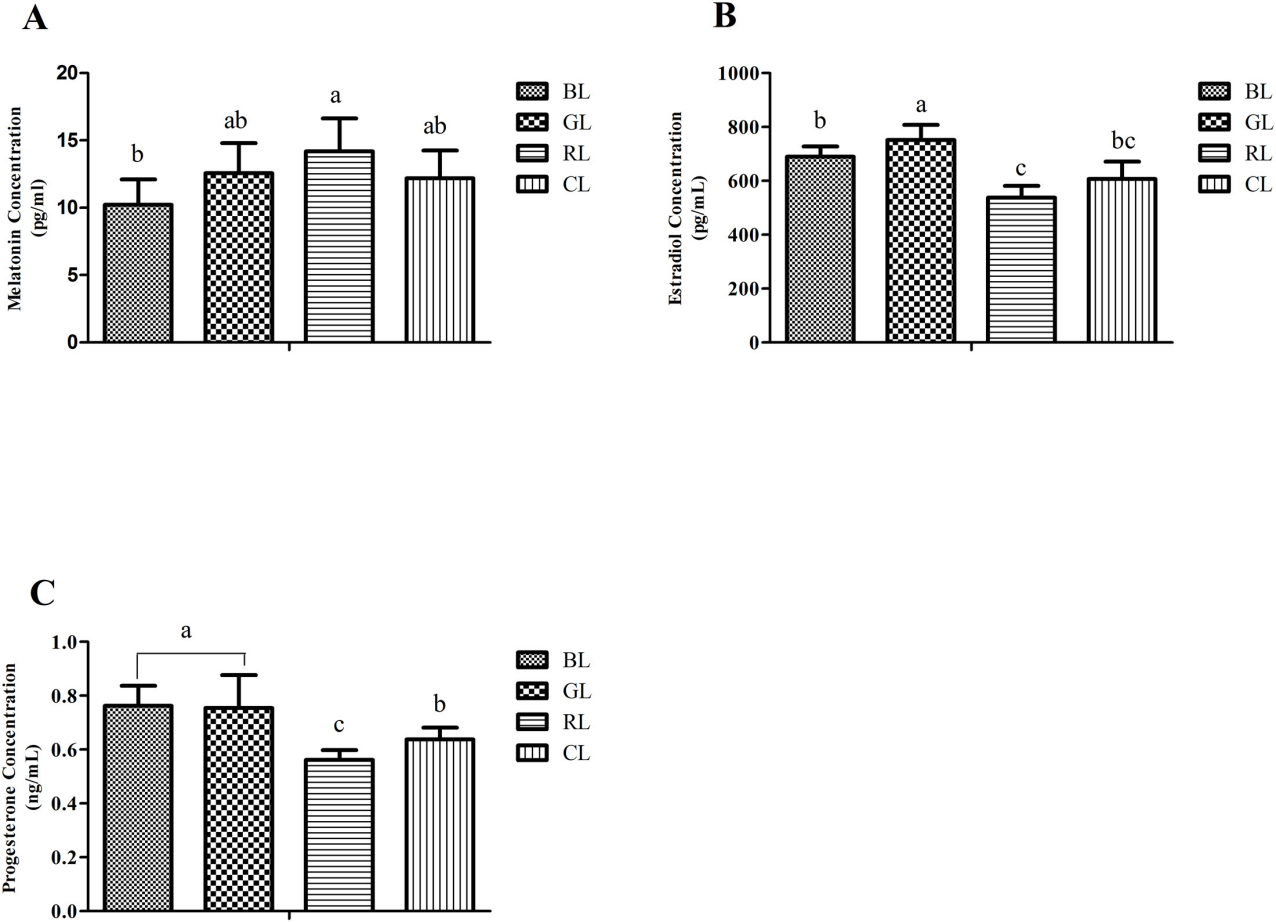
Effect of monochromatic light on plasma concentrations of melatonin, estradiol and progesterone. Results are expressed as mean ± SD (n = 5). BL = blue light, GL = green light, RL = red light, and CL = cool white light (control group). Least square means with different letters are significantly different (*P* < 0.05).

The plasma concentrations of melatonin, estradiol and progesterone at 28 wk of age are summarized in Fig 2. Plasma melatonin was lowest in the BL laying hens (*P* < 0.05) (Fig 2A). Plasma estradiol was elevated in the GL laying hens, with the lowest estradiol in birds reared under RL (*P* < 0.05) (Fig 2B). GL and BL increased plasma progesterone relative to CL and RL at 28 wk of age (*P* < 0.05) (Fig 2C).

### Effects of monochromatic light on estrogen and progesterone receptor mRNA

**Fig 3:**
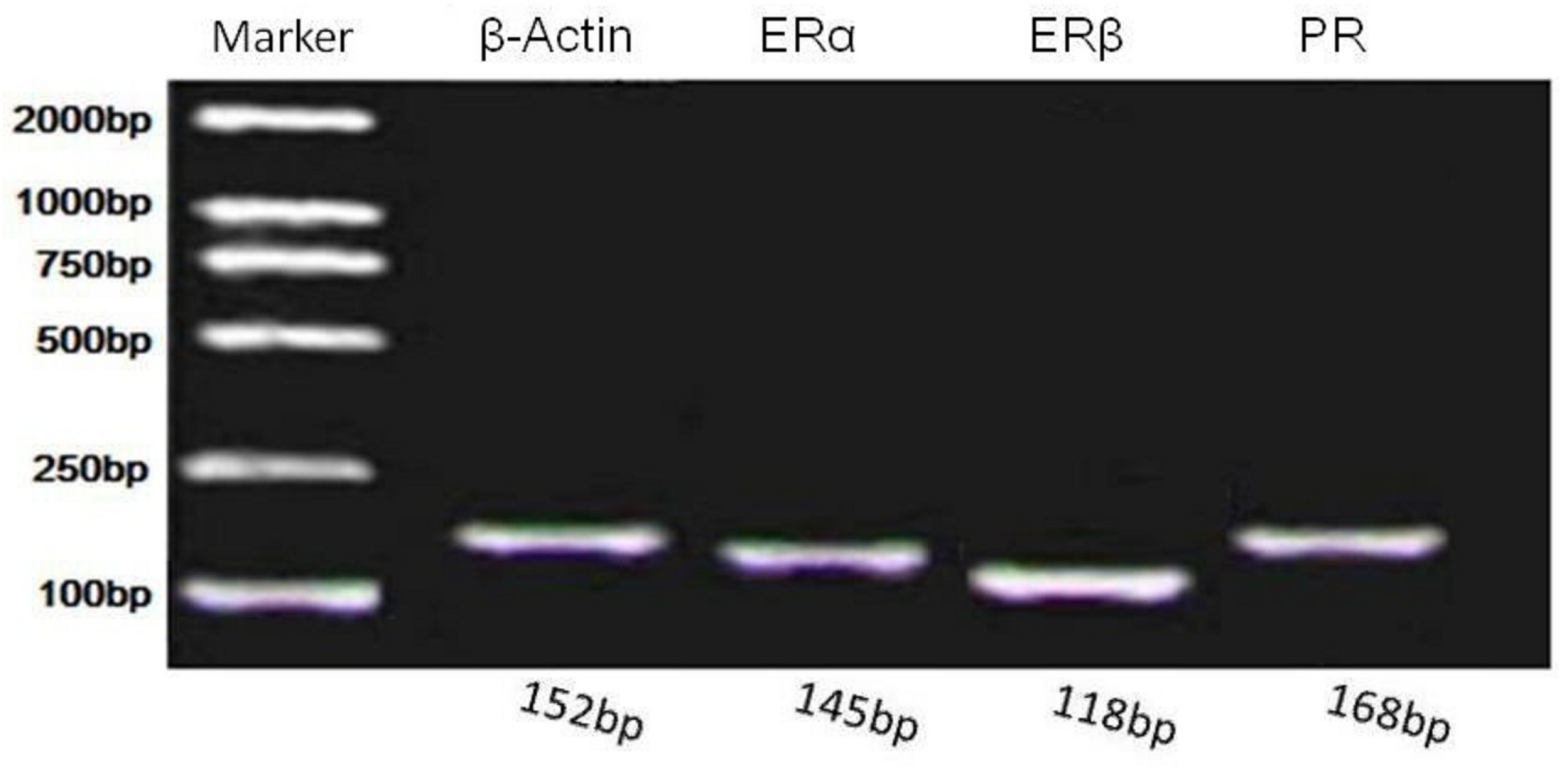
Gel image of PR, ERα and ERβ real time PCR products.

Real time PCR amplicon specificity for mRNAs encoding chicken β-actin, ERα, ERβ and PR in the granulosa layers of ovarian follicles were verified by gel electrophoresis (152, 145, 118, and 168 bp, respectively; Fig 3).

**Fig 4:**
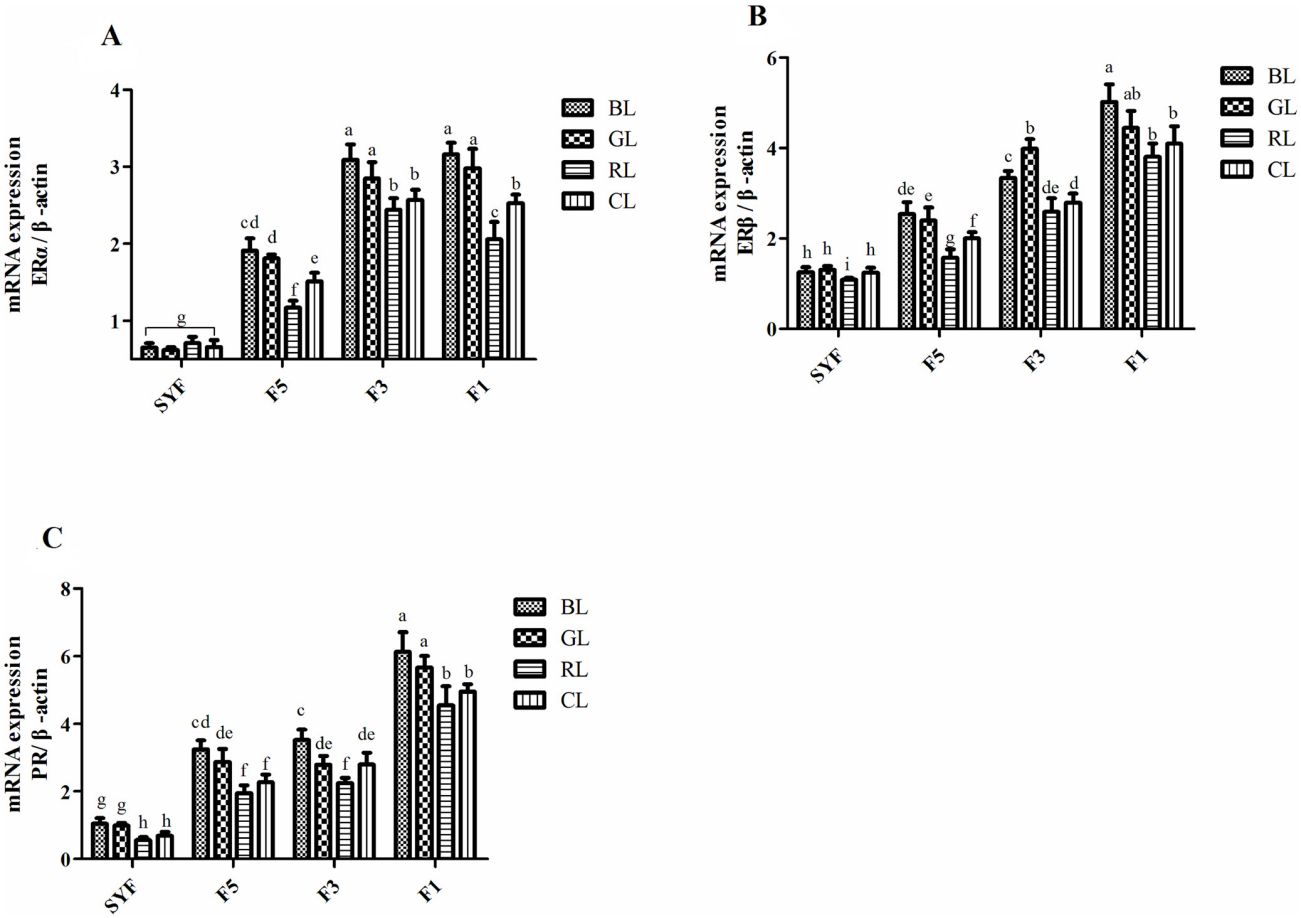
Effects of monochromatic light on ER isoforms and PR mRNA abundance in ovarian follicles of laying hens at 28 wk. (A) The abundance of ERα (B) ERβ, and (C) PR mRNA. Results are expressed as mean ± SD (n = 5). BL = blue light, GL = green light, RL = red light, and CL = cool white light (control group). ERα = estrogen receptor-α, ERβ = estrogen receptor-β, PR = progesterone receptor. SYF = small yellow follicle, F5 = the fifth largest preovulatory follicle, F3 = the third largest preovulatory follicle, and F1 = the largest preovulatory follicle. Least squares means with different letters are significantly different (*P* < 0.05).

The relative abundance of ERα, ERβ and PR mRNA in the granulosa layers of preovulatory follicles at 28 wk of age is shown in Fig 4. In the granulosa layers of preovulatory follicles in the CL group, the abundance of ERs mRNA was lowest in the SYF relative to other locations, and gradually increased from F5 to F1. There were no differences in ERα mRNA in the granulosa layers of SYF. In F5, F3 and F1, significant differences (*P* < 0.05) were observed in the relative abundance of ERα among lighting groups, with birds under BL and GL having greater expression and birds under RL having relatively lower expression. The ERα mRNA abundance under BL was not different from GL. The relative abundance of ERβ (Fig 4B) and PR mRNA (Fig 4C) showed patterns that were similar to ERα except that monochromatic light affected mRNA expression of ERβ and PR in the granulosa layers of F5 (*P* < 0.05). Treatment with BL resulted in increased expression of ERβ mRNA in granulosa layers of F5, F3 and F1, while GL increased ERβ mRNA in F5 and F3, relative to other lighting conditions (*P* < 0.05). Treatment with BL increased expression of PR mRNA in all of the granulosa layers of follicles (*P* < 0.05), treatment with GL increased expression of PR mRNA in granulosa layers of SYF, F5 and F1 (*P* < 0.05), and treatment with RL inhibited expression of PR mRNA in F3 (*P* < 0.05), relative to other lighting conditions.

### Effects of monochromatic light on protein expression of estrogen and progesterone receptors

**Fig 5:**
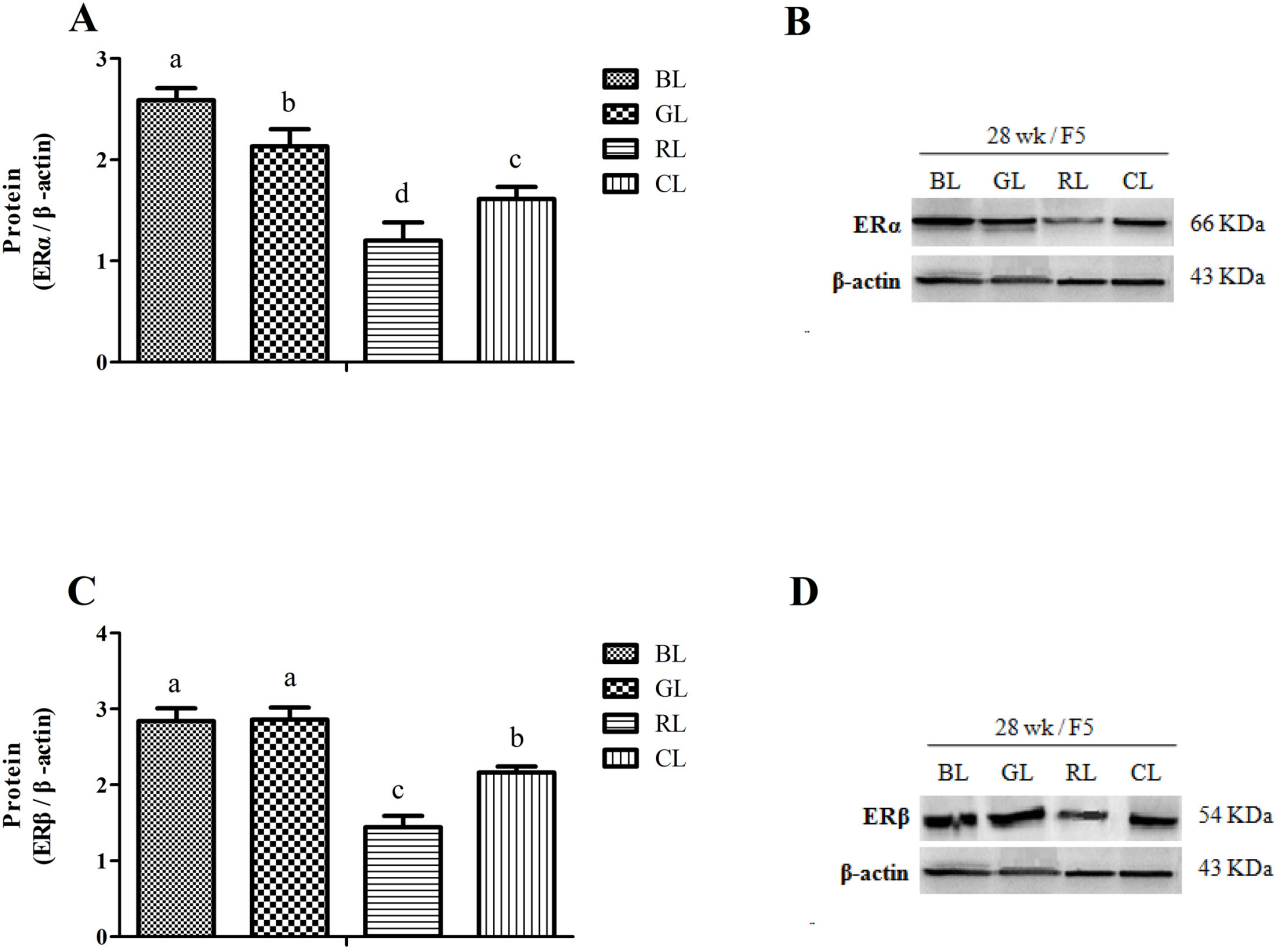
Effects of monochromatic light on ER isoform protein content in F5 of laying hens at 28 wk. (A) Protein abundance of ERα and (C) ERβ. Proteins detected by western blot from the chicken F5 were quantified by densitometric analysis and normalized to β-actin protein content. (B) Representative blots of ERα and β-actin, and (D) ERβ and β-actin. Results are expressed as mean ± SD (n = 5). BL = blue light, GL = green light, RL = red light, and CL = cool white light (control group). ERα = estrogen receptor-α, ERβ = estrogen receptor-β. Least squares means with different letters are significantly different (*P* < 0.05).

Because mRNA was most affected in F5, we evaluated the ER isoforms at the protein level in F5. On the blots, two bands, 66 and 54 KDa, corresponding to ERα and ERβ were detected (Fig 5B and 5D). Consistent with ERs mRNA, protein abundance of ERα (Fig 5A) and ERβ (Fig 5C) were influenced by monochromatic light (*P* < 0.05). There was greater ERα or ERβ protein in BL and GL than CL, and lowest amounts of protein in the RL group.

**Fig 6:**
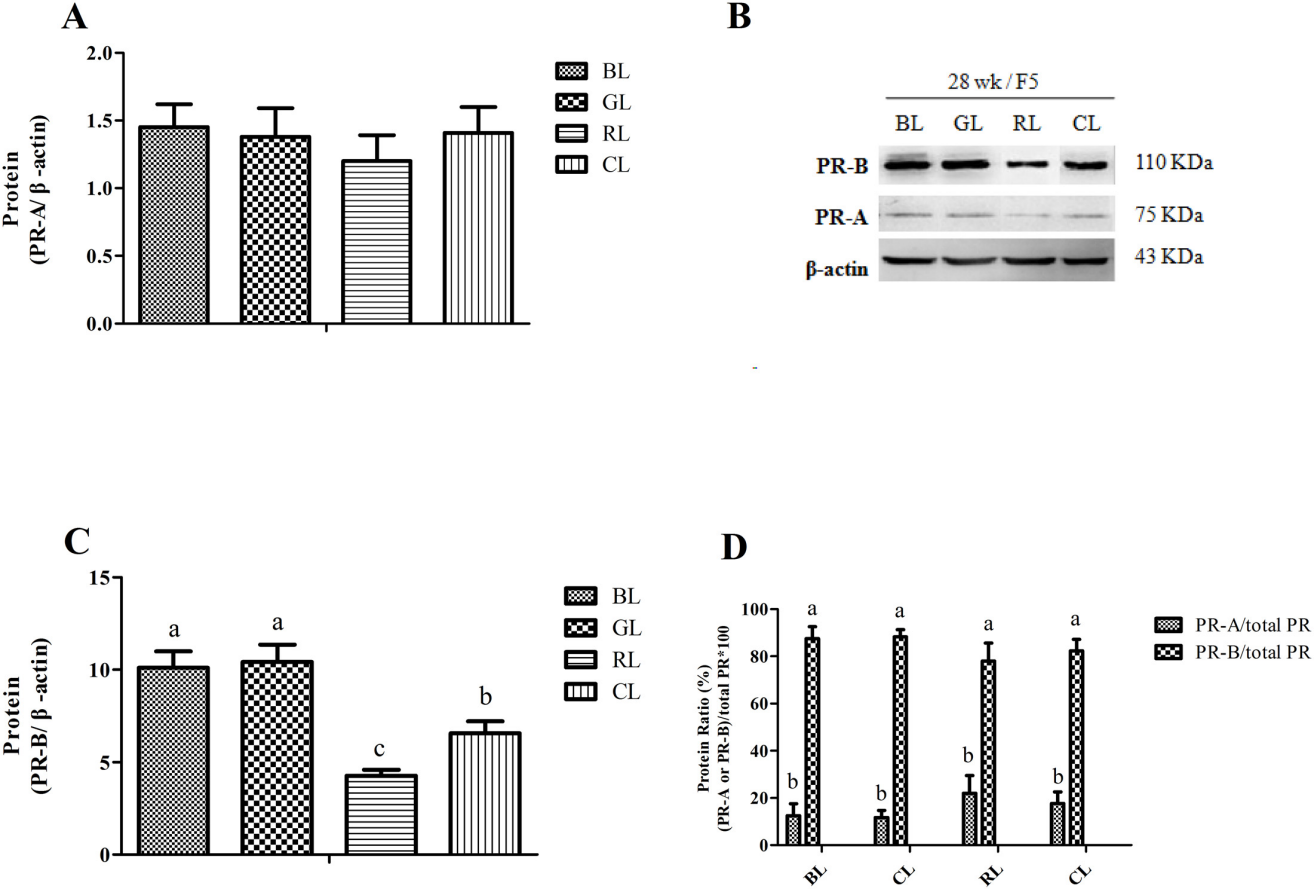
Effects of monochromatic light on PR isoform protein content in the F5 of laying hens at 28 wk. (A) Protein abundance of PR-A and (C) PR-B. Proteins detected by western blot from the chicken F5 were quantified by densitometric analysis and normalized to β-actin protein content. (B) Representative blot of PR-A, PR-B, and β-actin. (D) PR-A or PR-B ratio to total protein abundance (PR-A+PR-B) in the four light groups. Results are expressed as mean ± SD (n = 5). BL = blue light, GL = green light, RL = red light, and CL = cool white light (control group). Least square means with different letters are significantly different (*P* < 0.05).

PR isoform protein in the granulosa layers of F5 was also measured. Three bands, 75, 110 and 43 kDa, corresponding to PR-A, PR-B and β-actin, respectively, were detected in all blots (Fig 6B). Results showed that monochromatic light had no effect on the expression of PR-A protein (Fig 6A). However, BL and GL treatments increased PR-B in F5 (*P* < 0.05) in contrast to the RL treatment that reduced PR-B protein (*P* < 0.05) (Fig 6C). We used the ratio of PR-A or PR-B to total PR (total PR = PR-A+PR-B) as a measure of PR mRNA translational efficiency (Fig 6D). The PR-B/PR was greater than PR-A/PR in all light treatment groups. The PR-B/PR (control group, 82.33%) in the granulosa layers of F5 increased under BL (87.46%) and GL (88.31%), but was diminished in hens reared under RL (78.06%).

## Discussion

### Egg production traits

The hypothalamic photoreceptors are more sensitive to blue and green than red light when directly illuminated, although red light better penetrates the photoreceptors [2, 30–32]. It is known that different wavelengths of light affect egg production traits. In the present study, birds reared under BL increased EN300. Meanwhile, there was a trend for EN300 to increase in the GL group. Lauber et al [33] demonstrated that green and blue lights promoted chicken growth and reproduction processes. Er et al [10, 11] showed that egg production and feed conversion rate under blue light and the egg quality under green light were ideal for rearing Hy-Line Brown hens. These findings are consistent with the present study.

Birds have maximum sensitivity in a similar part of the light spectrum as humans (545–575), thus the retinal photoreceptors are most sensitive to green light [30]. Chickens reared under GL started the peak laying period a week earlier, with the following rank order of lighting effects: GL > BL > CL > RL. This finding corroborates Er et al [11].

It was suggested that red light might cover the shortage of sensitivity for the hypothalamic photoreceptors via increasing numbers of red light photons that penetrate into hypothalami [2]. Those data combined with the present results suggest that the egg-laying rate of BL chickens during the peak laying period was greater than other groups, although the RL group was not different from the GL and control group.

### Plasma concentrations of gonadal hormones

Melatonin is important hormone signal during illumination influencing chicken reproductive system [16]. Hypothalamus is the highest nerve centre for the hypothalamic pituitary gonadal (HPG) axis. Some studies indicated that melatonin acted directly on hypothalamus and inhibited secretion of Gonadotropin-releasing hormone (GnRH)[34, 35]. And Martin and Klein suggested that melatonin inhibited release of follicle-stimulating hormone (FSH) and luteinizing hormone (LH) in pituitary [36]. But the detailed molecular mechanism is complicated and still not clear now. However, we inferred that low level melatonin in the BL laying hens could activate the hypothalamic pituitary gonadal (HPG) axis.

Unlike mammals, the main sources of estrogens in chickens are theca cells, whereas for progesterone it is cells of the granulosa layer [37–40]. In birds, estrogen is critical for gonadal differentiation and development, reproductive behaviour, egg white protein synthesis in the oviduct, synthesis of egg yolk proteins in the liver, and mobilization of calcium for egg shell formation [15, 41], and estradiol is the predominant circulating form of estrogen [42]. In our study, the secretion of estradiol was enhanced by GL at 28 wk. Progesterone regulates reproductive functions such as uterus endometrium decidualization, maintenance of pregnancy and protection of the developing embryo and fetus [43, 44]. Our data demonstrate that BL and GL induced secretion of progesterone at 28 wk. These results support the idea that the secretion of LH and FSH were enhanced by blue and green light from 19 to 36 wk [45], and that LH and FSH regulate secretion of gonadal hormones. Furthermore, monochromatic light was also shown to affect the expression and secretion of GnRH in the hypothalamus [46], and GnRH could stimulate secretion of LH and FSH in pituitary. According to these studies, we inferred that monochromatic light affect estrogen and progesterone expression via regulating secretion of GnRH, LH, and FSH.

### ER mRNA and protein abundance in different tissues

Estrogen also exerts its actions by binding to nuclear estrogen receptors that function as ligand-activated transcription factors. In this study, we found the greatest expression of ERα and ERβ mRNA in the granulosa layer of F1, intermediate expression in F3 to F5, and the lowest expression in SYF, consistent with Hrabia et al [41]. The results confirm that there is expression of both ERα and ERβ mRNA in all granulosa layers of chicken follicles, suggesting that both ERα and ERβ have biological functions related to estrogen action in chicken follicles.

Treatment with BL increased expression of ER mRNA in granulosa layers F5, F3 and F1, while treatment with GL increased expression of ERα mRNA in granulosa layers F5, F3 and F1 and ERβ mRNA in granulosa layers F5 and F3. In addition, treatment with RL inhibited expression of ERα mRNA in granulosa layers F5 and F1, and ERα mRNA in granulosa layers SYF and F5. Western blots confirmed that these changes were accompanied by similar changes in the amount of protein in the granulosa layer F5. F5, as a dominant follicle, is sensitive to steroid stimulation during the rapid growth phase [47]. Earlier studies suggested that monochromatic light may affect egg quality [48] [10], pullet mortality [9], testosterone secretion and myofiber growth [14], but studies of the effects on production and gene expression are lacking. Jin et al. showed that monochromatic light altered expression of arylalkylamine N-acetyltransferase (AANAT) mRNA in chick pinealocyte and retinal cells [21], and Li et.al demonstrated that monochromatic light regulated expression of melatonin receptor subtypes (Mel 1a and Mel 1c) in broiler bursas [49]. These results support the present study where by monochromatic light affected expression of hormone receptor genes closely related with follicle development in laying hens.

### PR mRNA and PR-A/B protein abundance in different tissues

The biological effect of progesterone on target cells is elicited through progesterone receptor (PR)[44]. We detected PR mRNA in all granulosa layers of chicken follicles with the greatest expression in F1. The presence of the transcript suggests that PR plays a role in development and growth of follicles, particularly for F1. The findings are similar to previous studies showing that PR was necessary for ovulation [50]. In present study, treatment with BL resulted in increased expression of PR mRNA in all granulosa layers of follicles, while treatment with GL increased expression of PR mRNA in granulosa layers SYF, F5, and F1. Both estrogen and progesterone can regulate expression of PR, and in most target tissues estrogen increased the amount of PR [51, 52]. Moreover, estradiol and progesterone quantities were enhanced by blue and green light. These findings suggested that monochromatic light affected expression of PR mRNA via regulating secretion of estradiol and progesterone.

Both PR isoforms were detected in the F5 granulosa layer; monochromatic light affected expression of PR-B but not PR-A. This indicates different pathways for PR function in granulosa layers of chicken follicles under stimulation of monochromatic light. There was also a difference between PR-A and -B in their ratio to total PR protein. A greater amount of PR-B in proportion to total PR suggests that this PR isoform is more involved in the modulation of granulosa layer function in the chicken ovary. There were also changes in PR-B protein that confirmed the effects of monochromatic light on expression of PR mRNA. In particular, BL and GL enhanced PR-B/total PR. These data indicate that PR-B is more involved than PR-A in the modulation of granulosa layer activity in response to monochromatic light. These conclusions are also supported by previous studies that showed that there was more PR-B than PR-A expression in the chicken ovary and that this difference persisted after estrogen treatment [44]. PR has been considered as the key signaling molecule to mediate wall rupture in ovarian follicle [50]. In our study, we inferred that the increase expression of PRs promoted follicle wall rupture.

Hence, we can describe a possible mechanism for how blue and green light affect egg production traits in laying hens. Birds reared under blue and green light activated the hypothalamic pituitary gonadal (HPG) axis and increased estrogen and progesterone, and the increase in hormone production enhanced egg production traits through regulation of hormone receptors. Through identification of molecular mechanisms about monochromatic light regulating reproductive system in chickens, this research has the potential to increase egg laying traits with monochromatic light in laying hens, a major economic concern in the poultry industry. In future research, we will focus mainly on investigating the detailed mechanisms.

In the present study, we demonstrated the effect of monochromatic light on egg production traits, plasma melatonin, estradiol and progesterone, and expression of three mRNAs and four proteins that are involved in follicle development in laying hens. These results indicate that blue and green monochromatic lights promote egg production traits via stimulating gonadal hormone secretion and expression of hormone receptors. The changes in PR-B protein suggest that this form of progesterone receptor plays a predominant role in mediating progesterone action in the granulosa layers of preovulatory follicles in chickens in response to monochromatic light.

## Supporting Information

S1 Table Real Time PCR primer sequences of *ERα*, *ERβ* and *PR*. (DOCX)

S2 Table The effect of monochromatic light on EN300, PLP and Laying Peak in PLP. (DOCX)
