# Supplementary material for: Effect of Monochromatic Light on Expression of Estrogen Receptor (ER) and Progesterone Receptor (PR) in Ovarian Follicles of Chicken
Source: PLoS One. 2015 Dec 1;10(12):e0144102. doi: 10.1371/journal.pone.0144102 (PMC4666490; doi:10.1371/journal.pone.0144102)
Supplement: S1 Table — (DOCX) [file pone.0144102.s001.docx]

**S1 Table. Real Time PCR primer sequences of** ***ERα*, *ERβ* and *PR*.**

| Gene^1^ | Primer sequence (5'-3')^2^ | Product Length (bp) | Accession No. |
| --- | --- | --- | --- |
| *β-actin* | F: GAGAAATTGTGCGTGACATCA | 152bp | NM_205518.1 |
|  | R: CCTGAACCTCTCATTGCCA |  |  |
| *ERα* | F: TATTGATGATCGGCTTAGTCTGGC | 145bp | NM_205183.2 |
|  | R: CGAGCAGCAGTAGCCAGTAGCA |  |  |
| *ERβ* | F: CTCAGCACAGTCAGTCCAGAACA | 118bp | NM_204794.2 |
|  | R:TCAGGGACATCATCATGGAGG |  |  |
| *PR* | F: ATCATCGTTCTATTCACTGT | 168bp | [NM_205262.1](http://www.ncbi.nlm.nih.gov/entrez/viewer.fcgi?db=nucleotide&id=45383981) |
|  | R: CTCGTTCTCATCTCATCAA |  |  |

Note:1 *ERα*= Estrogen receptor-α, *ERβ*= Estrogen receptor-β, *PR=* Progesterone receptor.

^2^ F = forward, R = reverse.
