# Supplementary material for: Effect of Monochromatic Light on Expression of Estrogen Receptor (ER) and Progesterone Receptor (PR) in Ovarian Follicles of Chicken
Source: PLoS One. 2015 Dec 1;10(12):e0144102. doi: 10.1371/journal.pone.0144102 (PMC4666490; doi:10.1371/journal.pone.0144102)
Supplement: S2 Table — (DOCX) [file pone.0144102.s002.docx]

**S2 Table. The effect of monochromatic light on EN300, PLP**

**and Laying Peak in PLP.**

| Light source | BL | GL | RL | CL |
| --- | --- | --- | --- | --- |
| EN300 | 107.16±1.93^a^ | 104.41±1.94^ab^ | 100.01±1.89^b^ | 102.84±1.95^ab^ |
| PLP (week) | 26-34 | 25-33 | 28-33 | 26-33 |
| Egg-laying Rate in PLP(%) | 74.02±0.93^a^ | 72.00±0.93^b^ | 71.34±0.71^b^ | 72.35±1.02^b^ |

Note: Data are shown as least square means ± SD. Means with different superscript (a, b) in the same line for the same item differ significantly (p<0.05). EN300 = the total number of eggs at 300 days of age, PLP = peak laying period, Egg-laying Rate in PLP = egg-laying Rate in peak laying period. BL = blue light, GL = green light, RL = red light, CL= cool white light.
